# A community-based ultrasound determination of normal thyroid volumes in the adult population, Assin North District, Ghana

**DOI:** 10.11604/pamj.2020.37.251.20778

**Published:** 2020-11-18

**Authors:** Martin Tangnaa Morna, Derek Anamaale Tuoyire, Bashiru Babatunde Jimah, Ganiyu Adebisi Rahman, Sebastian Eliason, Anthony Baffour Appiah

**Affiliations:** 1 Department of Surgery, School of Medical Sciences, University of Cape Coast, Cape Coast, Ghana,; 2 Department of Community Medicine, School of Medical Sciences, University of Cape Coast, Cape Coast, Ghana,; 3 Department of Medical Imaging, School of Medical Sciences, University of Cape Coast, Cape Coast, Ghana,; 4 Ghana Field Epidemiology and Laboratory Training Programme (GFELTP), School of Public Health, University of Ghana, Legon, Accra, Ghana

**Keywords:** Determination, normal thyroid volume, Assin North District, Ghana, ultrasound

## Abstract

**Introduction:**

the purpose of this study was to measure thyroid volumes among normal sub-population of Ghanaians using ultrasonography in order to help provide preliminary local thyroid gland volume reference data for use in Ghana.

**Methods:**

this was a cross-sectional study in which the thyroid glands of 320 normal adults aged 18 to 95 years from six (6) communities in the Assin North District of Ghana were examined using ultrasonography. The volumes of the left and right lobes were summed to constitute the total thyroid volume. Information on socio-demographic characteristics and anthropometric parameters of subjects were also collected. The data were summarized using mean, standard deviation and proportions, whilst inferential analysis was done using the T-test, ANOVA test and Pearson correlation.

**Results:**

of the 320 adults examined, 284 (88.8%) were found to have normal thyroid glands. This consisted of 164 females and 120 males whose mean ages were 42.3 (±15.9) years and 45.4 (±15.9) years respectively. The overall mean total thyroid volume (MTTV) was 6.8±2.5 mL. This was greater in the males (7.1±2.7 mL) than in the females (6.6±2.2 mL). The MTTVs for three age groups; 30-39 years (7.1±2.1), 40-49 years (7.3±2.8 mL) and 50-59 years (7.1±3.0 mL) were greater than the overall MTTV. Thyroid volume had a positive correlation with body surface area (BSA) (r=0.119, p=0.046), but not with body mass index (BMI) (r=-0.021, p=0.719).

**Conclusion:**

this study estimated local reference values much lower than the WHO/ICCIDD thyroid volume reference values. This calls for the need for nationally representative studies to inform the establishment of standard local reference values for Ghana.

## Introduction

Despite over 20 years universal salt iodization campaign, thyroid disorders such as goiter remain important public health problems in Ghana [1]. Goitre is the most apparent manifestation of iodine deficiency [2]. Indeed, iodine deficiency is a major public health problem and is the leading preventable cause of mental impairment worldwide [3,4].

The diagnosis of goitre has traditionally been based on inspection and palpation of the thyroid. Based on such clinical examinations, goitre is said to be present when the lobes of the thyroid gland are larger than the terminal phalanges of the thumb of the person being examined [5]. Although clinical examination of the thyroid gland is simply performed with minimal cost, it is often difficult to precisely determine thyroid size as it depends to a large extent on the examiners and the characteristics of the study population [6,7]. In particular, it has been inadequate for distinguishing mild thyroid enlargement from normal [8].

Due to the inadequacy of clinical examination of the thyroid to accurately estimate thyroid size and volume, thyroid ultrasonography is increasingly being used for the assessment of thyroid size and volume. Ultrasonographic examination of the thyroid provides precise information on thyroid volume and structure and now is considered the most reliable method of determining thyroid size in population-based studies [6,9]. However, the use of ultrasonographic method for determination of thyroid size becomes effective, if reliable reference data exists for comparison. Regrettably, such standard reference data is still lacking for Ghana. Available reference values proposed by different scholars [6,10], including the WHO/ICCIDD recommended European reference values reported by Delange *et al*. [11] are fraught with discrepancies. A recent study done in Benin City in Nigeria to measure the volume of normal thyroid glands in children and compare the results with the WHO accepted values revealed that the local values derived were significantly lower than that of the internationally accepted [12]. These discrepancies have led to calls for the need to establish specific local reference values.

In view of this, the current study aims to measure the thyroid volumes of a sub-population of Ghanaians in order to help provide preliminary local thyroid gland volume reference data for comparison with reference values of the WHO, as well as other populations. The data will stimulate the need for scale-up assessment of thyroid volumes nationwide to establish a reliable reference for diagnosing goiter in the Ghanaian setting.

## Methods

**Study design:** a community-based cross-sectional design was employed to study the thyroid volume of 320 normal volunteers from six communities in the Assin North District of Central Region, Ghana.

**Study area:** Assin North District is one of the 20 administrative districts in the Central Region of Ghana with its capital at Assin Fosu. The district was chosen because the University of Cape Coast, School of Medical Sciences (UCCSMS) has adopted the six (6) communities within the district as a social laboratory to facilitate the training of medical students under the Community-Based Experience and Service (COBES) programme of the School of Medical Sciences. These communities include Breman, Dense, Aboteriyie, Ahuntamu, Assin Akyeano and Assin Kushea.

**Sample size and sampling technique:** the sample size was calculated using the formulae for estimating the sample size for a single population mean [13]:

$$n={\left(\text{Z}\sigma \right)}^{2}/e^{2}$$

Where n=minimum sample size, Z=value from the standard normal distribution of a specified confidence level, e=margin of error and σ is the population standard deviation. Studied literature showed that the standard deviation of mean thyroid volume of a normal population ranges from 0.3 to 12.0 [8,14-19]. To estimate the mean total thyroid volume of a normal population not exceeding the margin of error of 1.5 mL and at 95% confidence interval, we determined the sample size of 246. Factoring in a 10% non-response, a final minimum sample size of 271 was arrived at.

Proportionate allocation based on estimated population of 9576 (Breman 701, Dense 396, Aboteriyie 514, Ahuntamu 669, Assin Akyeano 1170 and Kushea 6126) was employed to allocate the sample size to each study community: Breman 19; Dense 11; Aboteriyie 15; Ahuntamu 19; Assin Akyeano 33; and Kushea 174. Temporary thyroid ultrasound center was set-up in each community and a consecutive sampling technique was used to recruit normal volunteers from different households. Exclusion criteria included participants with anterior neck swelling or clinical evidence of thyroid disease, smokers, persons on lithium, phenytoin, oral contraceptive drugs, and women during menstruation, pregnant women or women who had delivered within the last 12 months and persons with any systemic disorder.

**Data collection:** ethical approval was obtained from Institutional Review Board at the University of Cape Coast, Ghana (reference number: UCCIRB/EXT/2017/18) and volunteers who consented to participate in this study were recruited. All protocols involving informed consent were duly observed during and after this study. Participants were interviewed using a structured questionnaire to collect information on their demographic characteristics, history of salt and alcohol consumptions. A real-time ultrasound scanner (MEDISON SA8000SE-MAI, 1003 Dachi-Dong, Gangnam-Gu, Seoul Korea) with a 7.5 MHz, 50 mm linear transducer was used for thyroid examination. Thyroid examination was done by a radiologist with over 5 years´ experience in thyroid ultrasonography. Participants were examined while in a supine position with a hyper-extended cervical spine. Ultrasound gel was applied over the thyroid area with the transducer directly placed on the skin over the thyroid gland. Longitudinal and transverse scans were performed, to obtain length and width in centimeters of each thyroid lobe and isthmus.

The volume of one lobe of the thyroid was expressed in mL and estimated by the formula: volume of lobe=length × depth × width × π/6 [18]. The total thyroid volume was the summation of the volumes of left and right lobes excluding the volume of the isthmus [18]. Other anthropometric measurements done included: body mass index (BMI) (kg/m^2^), estimated as=weight kg/height (m)^2^ and body surface area (BSA) (m^2^) estimated as=weight (kg)^0.425^ x height (m)^0.725^ x 7184 x 10^-4^ [12,16].

**Data analysis:** the study recruited 320 normal volunteers but 36 participants with abnormalities were excluded after the thyroid ultrasound examination. The remaining 284, representing 88.8%, were included in the analysis. The data was captured and analyzed using SPSS IBM version 21. The normality of thyroid volumes measurement was confirmed with quantile-quantile (Q-Q) plot. Continuous variables such age, anthropometric and thyroid measurements were summarized using mean and standard deviation. The ages were categorized and expressed in proportions. For the purposes of comparison between sexes, the Student t-test was used for comparison in both sexes, while the Pearson´s correlation coefficient was used to relate the thyroid volume to height, weight and BSA. One way analysis of variance (ANOVA) was used to determine significant difference in thyroid measurement between the age groups. A significance level of p<0.05, was applied in statistical tests.

## Results

Two hundred and eighty-four (284) healthy volunteers aged 18-95 years including 164 (57.7%) females and 120 (42.3%) males with the mean age of 42.3 (±15.9) years and 45.4 (±15.9) years respectively were studied. The socio-demographic characteristics of healthy volunteers from the six communities are summarized in Table 1. Equal proportions of subjects (20.4%) were in the 20-29 and 30-39 year age groups. Nearly one-fourth (19%) of the respondents had no experience of formal education, with majority (62%) working as farmers. Almost all (96.1%) of the respondents often took iodized salt while 22.5% had a history of alcohol intake.

**Table 1:** socio-demographic characteristics of respondents in six communities, Assin North District

Characteristics	Frequency (n=284)	Percentage (%)
**Age group**		
<20	10	3.5
20-29	58	20.4
30-39	58	20.4
40-49	55	19.4
50-59	49	17.3
60-69	32	11.3
70+	22	7.7
**Gender**		
Male	120	42.3
Female	164	57.7
**Highest education level**		
None	54	19.0
Primary	36	12.7
JSS/JHS	109	38.4
SSS/SHS/vocation	53	18.7
Tertiary	32	11.3
**Occupation**		
Public servant	13	4.6
Artisan	29	10.2
Trader	18	6.3
Farmer	176	62.0
Other	12	4.2
Unemployed	25	8.8
Student	11	3.9
**Marital status**		
Single	65	22.9
Co-habiting	13	4.6
Married	158	55.6
Divorce	26	9.2
Widow	22	7.7
**Daily salt intake**		
Often	273	96.1
Not often	11	3.9
**Alcohol intake**		
Yes	64	22.5
No	173	60.9
No response	47	16.5

JSS: junior secondary school; JHS: junior high school; SSS: senior secondary school; SHS: senior high school

In Table 2, the distribution of anthropometric measurement and thyroid volumes by sex of respondents are summarized. The mean weight, BMI and BSA were slightly higher in women compared to men in contrast to height, but only mean height and BMI were statistically different by sex (at p<0.001). The mean total thyroid volume was 6.8 mL, which was greater in males than females (men: 7.1 ml, women: 6.6 ml). The right lobe was slightly larger than the left lobe in the total sample (right: 3.4 ml, left: 3.2 ml) and in men (right: 3.7 ml, left: 3.1 ml) and differed significantly between both sexes (p=0.030). The mean thickness of the isthmus was 0.3 ml, which differed slightly between both sexes (p<0.043). The correlation of five parameters (age, weight, height, BSA and BMI) with thyroid and isthmus volumes are summarized in Table 3. The height and BSA were positively correlated with the total volume, left lobe and isthmus volumes. There was no significant correlation between BMI and thyroid and isthmus volumes.

**Table 2:** distribution of anthropometric measurement and thyroid volumes by sex of respondents in six communities, Assin North District

Variable	Total sample (n=284)	Males (n=120)	Females (n=164)	p-value
**Anthropometric measurement**				
Weight (Kg)	59.0±11.9	58.6±9.4	59.3±13.4	0.300
Height (m)	1.6±0.1	1.7±0.1	1.6±0.7	0.001
BMI (Kg/m2)	22.8±4.9	20.7±3.0	24.3±5.5	0.001
BSA (m2)	1.6±0.3	1.6±0.3	1.6±0.4	0.189
**Thyroid volumes**				
Left lobe (mL)	3.2±1.4	3.1±1.3	3.3±1.5	0.125
Right lobe (mL)	3.4±1.1	3.7±1.1	3.3±1.1	0.030
Total volume (mL)	6.8±2.5	7.1±2.7	6.6±2.2	0.073
Width of Isthmus (m)	0.3±0.1	0.3±0.1	0.3±0.1	0.043

**Table 3:** correlation between age, anthropometric parameters and thyroid volumes and isthmus thickness of respondents in six communities, Assin North District

Variable	Left lobe		Right lobe		Total volume		Isthmus	
	r	p	r	p	r	p	r	p
Age (years)	-0.061	0.307	-0.034	0.564	-0.009	0.886	-0.083	0.164
Weight (kg)	0.068	0.257	0.093	0.118	0.097	0.103	0.110	0.063
Height (m)	0.060	0.313	0.186*	0.002	0.205**	0.001	0.174*	0.003
BSA (m^2^)	0.073	0.220	0.113	0.058	0.119*	0.045	0.131*	0.028
BMI (kg/m^2^)	0.024	0.687	-0.015	0.805	-0.021	0.719	-0.002	0.976

r-coefficient and p-significant value (2-tailed) at **p<0.01 or *p<0.05

The mean, standard deviation and p-value from ANOVA test of various thyroid dimensions (width, thickness and height) for right and left lobes and total volume by age of participants are displayed in Table 4. The mean total thyroid volume (6.8±2.5 mL) was lower than those recorded among the age brackets: 30-39 years (7.1±2.1), 40-49 year (7.3±2.8 mL) and 50-59 years (7.1±3.0 mL). The mean dimensions of both left and right lobes were not significantly different among the various age brackets (at p>0.05), however, the total volume differs significantly across the age brackets (p=0.046). In Table 5, the findings on normal total thyroid volumes in reviewed literature are summarized.

**Table 4:** mean and standard deviation (SD) of thyroid volumes by age groups of respondents in six communities, Assin North District

Age	Number	Left lobe (mean ±SD)					Right lobe (mean ±SD)			Total vol. (mean ±SD)
		Width (mm)	Thickness (mm)	Height (mm)	Volume (mL)	Width (mm)	Thickness (mm)	Height (mm)	Volume (mL)	
<20	10	1.4±0.2	1.4±0.8	3.5±0.3	3.6±1.9	1.6±0.6	1.2±0.2	3.4±0.9	3.4±1.2	6.7±2.5
20-29	58	1.5±0.3	1.2±0.2	3.2±0.6	3.0±1.1	1.5±0.3	1.2±0.2	3.2±0.4	3.3±1.0	6.2±1.8
30-39	58	1.5±0.4	1.2±0.3	3.3±0.6	3.4±1.4	1.5±0.3	1.3±0.5	3.3±0.3	3.5±0.9	7.1±2.1
40-49	55	1.5±0.3	1.3±0.3	3.4±0.4	3.4±1.1	1.6±0.4	1.3±0.5	3.4±0.3	3.6±1.1	7.3±2.8
50-59	49	1.5±0.4	1.2±0.4	3.3±0.6	3.5±1.7	1.6±0.5	1.3±0.4	3.4±0.3	3.5±1.3	7.1±3.0
60-69	32	1.5±0.5	1.1±0.4	3.2±0.7	2.8±1.4	1.5±0.4	1.3±0.2	3.2±0.5	3.5±1.1	6.6±2.6
70+	22	1.4±0.3	1.2±0.3	3.0±0.8	2.7±1.6	1.5±0.4	1.2±0.2	3.0±0.4	2.9±0.9	5.7±5.2
Total	284	1.5±0.3	1.2±0.3	3.3±0.6	3.2±1.4	1.5±0.4	1.3±0.4	3.5±0.4	3.4±1.1	6.8±2.5
p-value		0.853	0.138	0.093	0.054	0.599	0.255	0.056	0.110	0.046^*^

* p-significant value (2-tailed) from the ANOVA test of significant difference at p<0.05; volume-vol

**Table 5:** summary of reviewed literature on normal thyroid volumes across continents

Author, year	Sample size	Sex	Age range (years)	Thyroid volume (mL) ± SD	Country/ continent
Current study, 2019	284	120 M	18-95	6.8±2.5	Ghana
		164 F			
WHO/ICCD, 1997	7599	3758 M	7-15	16.1	Europe
		3841 F			
Yousef, 2011	103	75 M	19-29	6.44±2.44	Sudan
		28 F			
Seker, 2010	251	105 M	15-78	13±6.27	Turkey
		146 F			
Ivanac, 2004	51	-	20-38	10.68±2.83	Croatia
Chanoine, 1991	256	-	0-20	11.6±4.4	Belgium
Turcios, 2015	100	21 M	18-50	6.6±0.3	Cuba
		79 F			
Kayastha, 2010	485	221 M	1-83	6.63±2.50	Nepal
		264 F			
Brahmbhatt, 2000	118	90 M	6-15	23±12	Baroda, India
		28 F		25.0±9.6	
	412	193 M	6-15	29±9	Dang, India
		219 F		27.4±11.5	

ICCIDD-international council for the control of iodine deficiency disorders

## Discussion

In the twenty-first century, the World Health Organization introduced a new diagnostic criterion for goiter [20]. The diagnosis of goitre which used to be based on palpation is now based on volume measurement using ultrasonography [15,19,21]. Volume measurement of the thyroid gland has become easy and quick to obtain because the gland has a different echogenicity compared with associated soft tissues [15]. This technique may be expensive but remains the gold standard for measuring thyroid volumes. Our study on thyroid volume in normal adults in Assin North District of Ghana provides preliminary data on local reference values with which any adult with suspect thyroid anomalies can be compared. In accordance with WHO/ICCDD recommendation for measuring thyroid volumes, our study assessed the total thyroid volume by summing up the volumes of left and right lobes, excluding the size of the thyroid isthmus [8,9,15,17,19,22,23].

**Total thyroid volume:** we compared our mean normal total thyroid volume with reviewed literature (Table 5). In this study, the mean total thyroid volume was 6.8mL, a value comparable to the one reported from Sudan (6.44mL) [15], Cuba (6.6mL) [18] and Nepal (6.63mL) [17], among similar healthy adult populations using ultrasound. Despite the fact that previous studies have suggested an influence of socio-demographic and environmental factors (like dietary habits) on thyroid volume [11,12], they had no significant influence on the thyroid volume measured in this study and those in Sudan, Cuba and Nepal. The mean total volume was much lower than the one obtained in Turkey (13mL) [16] among adults and those from India (23-29mL) [8] and Belgium (11.6mL) [14] among younger subjects. Even the WHO/ICCIDD recommended reference of 16mL for young people (children 15 years and below) [11] was higher than our reported mean. This variation in findings may be attributed to differences in assessment of thyroid volumes, skewness in the age distribution of participants (mostly children under 15 years) and approaches used in selecting subjects. In addition, Ghana has promoted the campaign on iodized salt consumption for over 20 years, which may explain the lower mean total thyroid volume compared to other studies [8,14,16,19].

In contrast with the findings that thyroid volume (TV) increases with advancing age, we found no significant association between age of adult subjects and TV [11,12]. This may be that most adult thyroid glands have reached their optimum sizes compared to children who have a proportional effect of age on the TV until puberty [11]. Our findings, 7.1mL in 120 males and 6.6mL in 164 females, are lower than the reported 15.87mL in 105 males and 10.94mL in 146 females in Turkey [16]. But similar variation in mean thyroid volume between males and females was observed in both studies. A different observation was made by Yousef *et al*. among 103 normal Sudanese [15], the observed differences in reports could be explained using the differences in male to female ratio, age distribution and ethnicity/race within the study populations [15,16].

Generally, the right lobe is considerably higher than the left lobe [17,22]. Consistent with the established fact [17,22] and previous research, the present study found the mean volume of right lobe slightly larger than the left lobe. A similar observation was made by Yousef *et al*. in 103 Sudanese [15], Ivanac *et al*. in 51 Croatian [19] and Kayastha *et al*. in 485 Nepalese [17]. Our estimate for right and left thyroid lobes were slightly higher compared with similar studies in Africa by Yousef *et al*. [15], WHO reported 3.38 (1.37) mL and 3.09 (1.24) mL for right and left lobe respectively. No physiologic or socio-cultural explanation has been assigned to this variation in volumes of the left and right lobes [17].

**Age-specific thyroid volume:** studies have positively correlated age with TV [14,17], but our age-specific TVs did not increase with increasing age. The study conducted by Chanoine *et al*. and colleagues in 256 euthyroid individuals, revealed that thyroid volume significantly increased (p<0.001) until the age of 8 without being influenced by sex and thereafter varied widely by sex [14]. The observed variations in mean TVs (p=0.046) among our age groups confirm the above statement, despite the fact adult thyroid glands do not increase considerably with age. We agreed that the observed mean TVs in various age brackets were modified by sex and not solely advancing age [11].

**Thyroid volume and sex:** despite the observed variation in mean total thyroid volume among sexes in this study, there was no significant difference in mean total thyroid volume between males and females (p=0.073). Similar observations were made by Kayastha *et al*.in Nepal [17], Turcios *et al*. in Cuba [18] and Brahmbhatt *et al*. in Baroda, India [8]. In contrast, Brahmbhatt *et al*. conducted similar studies among 412 normal children in Dang, India and this time observed a significant difference in mean total volume between both sexes (p=0.003) [8]. Hegedus *et al*. also found a significant difference in mean total thyroid volumes between males (19.6±4.7 ml) and females (17.5±4.2 ml) (p<0.001) among 271 healthy subjects of ages 13-91 years [23]. Studies have associated the observed variation with body size in terms weight and height between males and females [17,23]. Possible factors such pregnancy and menstruation [15], that contribute to increasing thyroid volume in women were adjusted during the design stage of this study. Hence, the observed variation that existed between our findings and previous studies [8,23].

**Thyroid volume and anthropometric parameters:** several studies have recognized some key anthropometric parameters (weight, height, BMI and BSA) for predicting thyroid volume [6,8,15,18,23,24]. Şeker *et al*. report a positive correlation between thyroid volume and BSA, BMI, height and weight (p<0.001) among Turkeys´ adults of age range between 15 and 78 years [16]. Our findings confirmed a positive correlation between thyroid volume and height and BSA, but not BMI and weight. Our findings were in accordance with previous observations made by Turcios *et al*. who found that the total thyroid volume increased with increasing BSA and not BMI among adults of ages 18 to 50 years [18]. Brahmbhatt *et al*. made very different observations in two Indian subpopulations (Baroda and Dang) of ages 6 to 15 years, they observed no significant association between total thyroid volume and the key anthropometric parameters (weight, height, BMI and BSA) [8]. Our findings were in accordance with the WHO/ICCIDD report which indicated thyroid volume as a function of BSA, irrespective of age [11].

**Limitations of the study:** although this study involved a larger number of subjects than previous studies [11,15,16,18], it does not give an accurate picture of central region as a whole as only six communities in one district out of 26 districts in the region were studied. Also, the findings of this study are not representative of Ghana, as only one region out of sixteen regions in Ghana were involved. This study did not cover the entire region or Ghana due to resource constraints. However, our objectives of providing preliminary local reference data on thyroid volumes to establish the existence of differences with WHO/ICCIDD data to influence stakeholder discussion and further assessments have been achieved.

## Conclusion

In this study, we obtained thyroid volume similar to values reported in Sudan, Nepal, Cuban, but lower than the WHO/ICCIDD thyroid volume references and other previous studies. Generally, thyroid volumes increased with age, and were larger in men than in women. The volume of the right lobe of the thyroid gland is slightly higher than the left lobe in both sexes. The relation between BSA and thyroid volume is consistent with what is documented. This study has provided a preliminary local reference value of thyroid volume, we recommend further studies on a national scale to established standard national reference value of normal thyroid volume in Ghana.

### What is known about this topic

- Reported thyroid volumes in Africa differ from WHO/ICCIDD recommended reference values;
- Generally, thyroid volumes increase with advancing age and greater in females than males;
- Body surface area (BSA) and body mass index (BMI) influence the size of thyroid volume.

### What this study adds

- The thyroid volume of the Ghanaian subpopulation is lower than WHO/ICCIDD recommended reference value for diagnosing thyroid disorders and this confirmed previous studies;
- There is no significant increase in thyroid volume with advancing age in healthy adults but was greater in males than females;
- There is a significant positive correlation between thyroid volume and BSA.
